# Thirty years of ground deformation monitoring at Stromboli volcano

**DOI:** 10.1038/s41597-025-04850-y

**Published:** 2025-03-29

**Authors:** Valentina Bruno, Bellina Di Lieto, Salvatore Gambino, Mario Mattia, Danilo Messina, Laura Privitera, Pierdomenico Romano, Massimo Rossi

**Affiliations:** 1 Istituto Nazionale di Geofisica e Vulcanologia, Osservatorio Etneo, Catania, Italy; 2 Istituto Nazionale di Geofisica e Vulcanologia, Osservatorio Vesuviano, Napoli, Italy; 3 Università di Palermo, Dipartimento di Scienze della Terra e del Mare, Palermo, Italy; 4 Università di Catania, Dipartimento Scienze Biologiche Geologiche ed Ambientali, Catania, Italy

**Keywords:** Geophysics, Volcanology, Geodynamics, Natural hazards

## Abstract

Since 1992, INGV has collected tilt, GNSS (Global Navigation Satellite System) and strainmeter data, for the monitoring of effusive and explosive activity of Stromboli volcano. In this timespan, ground deformations related to eruptive activity, paroxystic explosions, cycles of inflation/deflation and many other phenomena of volcanological and geophysical interest have occurred. The dataset from the permanent geodetic networks spans the period 1992/2022 with a variable sampling rate, depending on the individual instrument: from the daily 3D coordinates of GNSS stations to the 1 Hz data of the strainmeter. The time series of GNSS, tilt and strainmeters data cover the entire spectrum of the volcanic and tectonic events observed at Stromboli. A GNSS-derived velocity field is also provided.

## Background & Summary

Stromboli, often referred to as the “Lighthouse of the Mediterranean” is one of the most active volcanoes worldwide. Its activity is characterised by continuous degassing accompanied by explosive transients of variable intensity. Occasionally, paroxysmal explosive activity interrupts the normal “strombolian” activity, increasing volcanic hazard for the numerous tourists and local population living at the base of the subaerial part of the volcano. Bevilacqua *et al*.^1^ compiled an historical catalogue of paroxysms that have occurred in the last ca. 140 years and Calvari *et al*.^2^ the eruptive activity recorded from 1879 to 2023. Eruptions occur frequently with lava flows, without any significant danger for population, reaching the sea, crossing the “Sciara del Fuoco” area, a large scarp in Stromboli’s NW flank. A major hazard is posed by the possible catastrophic collapses of the “Sciara del Fuoco” with the real possibility that tsunami waves could be generated as a result of the collapse (e.g.^3^), as happened, e.g., at Soufrière Hills Volcano^4^.

Because of the frequent and varied type of volcanic activity, Stromboli has been considered a “volcano laboratory” for centuries, where volcanologists and geophysicists from around the world come seeking to improve their knowledge on the mechanism of magma transfer from depth in open-conduit volcanoes, the relationship between structural and volcanological contexts, the morphologies related to magma flows and the flank dynamics in a volcanic area.

The INGV (Istituto Nazionale di Geofisica e Vulcanologia) has collected ground deformation data at permanent stations since 1992, transmitting the collected data to INGV headquarters in Catania and Naples. Tilt, GNSS and Strainmeter data, from 1992 to date, are one of the most important observations for the monitoring of the effusive and explosive activity of Stromboli.

Many eruptions and paroxystic explosions have been observed by our geodetic stations in these years, including cycles of inflation/deflation, precursors of dangerous explosions, the response of the volcanic plumbing system to tectonic activity, and a giant landslide that, in 2002, induced a tsunami that destroyed houses and docks^5^.

**Fig. 1:**
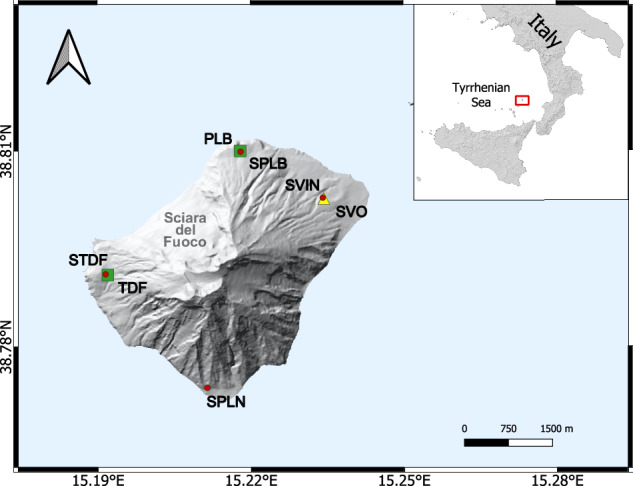
Maps of Stromboli volcano indicating the distribution of the monitoring geodetic instruments: tilt (green squares), GNSS (red circles) stations and strainmeter (yellow triangle). The inset on the top right indicates the location of Stromboli volcano in the Tyrrhenian Sea (Southern Italy).

We have re-processed, filtered and analysed the data from the three ground deformations monitoring networks (tilt, GNSS and Strainmeter; see Fig. 1 for the permanent network sites), with the intention of enabling the whole community of volcanologists and geophysicists to use our data for their research.

## Methods

GNSS stations, strainmeters and tiltmeters are the most widely used instruments for continuous monitoring of ground deformations in volcanic environments. The different instruments enable a multiparametric approach necessary to understand the ground deformation related to volcanic activity occurring at Stromboli, characterized by small variations, which are difficult to detect with a single method^6–16^.

Bubble tiltmeters installed a few meters from the surface can record ground tilts in the order of one part per ten million (10^−7^ radian or 0.1 µrad), whereas sophisticated long-base borehole tiltmeters can measure tilt changes up to one part per billion (10^−9^ radian or 10^−3^ µrad)^17^. Volumetric strainmeters (or dilatometers) installed on Stromboli volcano are of the Sacks-Evertson type. They can measure strain changes up to 10^−12^ over a wide range of frequencies up to 100 Hz^18–20^ and can resolve strain changes in the order of 5 × 10^−8^ over periods of several months. Both tiltmeters and strainmeters are capable of recording the whole spectrum of the elastodynamic deformation, from static to dynamic, with exceptionally high accuracy. Continuous Global Navigation Satellite System (GNSS) stations have improved the observation techniques in modern geodesy, becoming one of the most widespread techniques for observing ground deformations. The main advantage of GNSS measurements is the possibility to measure three-dimensional displacements and the long-term stability. This technique has subcentimetric precision in both horizontal and vertical components^21^. Tiltmeters and strainmeters have better accuracies than GNSS position estimates but, tilt and strain observations suffer from long-term drift and are more susceptible to site effects than GNSS observations. These, in fact, can be contaminated by atmospheric pressure, precipitation, tides, groundwater levels and deep pressure and temperature conditions^22,23^, nevertheless, both tilt and strain observations are useful in the band ranging from months to milliseconds. GNSS receivers are less sensitive but improve secular observations because of their greater spatial coverage and long-term stability^24^.

The combination of these observations thus provides a more comprehensive view of the volcanic processes.

### GNSS

#### GNSS network

The GNSS network was installed in Stromboli in June 1997. The first three stations (colocated with the tilt network) were Punta Labronzo (SPLB), Punta Lena (SPLN) and Timpone del Fuoco (STDF). The Trimble 4000 receivers collected data only for a few hours/day, due to limitations in power supply. Since 2003/2004, when the Trimble 4000 receivers were replaced with Leica 500 receivers, the stations have been able to collect data 24 hours/day. In 2003, a new station was set up in San Vincenzo COA (Centro Operativo Avanzato - SVIN), very close to the village. Since 2003, the data has been transmitted in real time at high frequency (1 Hz), through Wi-Fi networks and modems, to the INGV office in Catania. Here, the data are resampled at 30 seconds, archived and processed.

#### GNSS processing

The daily site coordinates were estimated from the GNSS data using the GAMIT/GLOBK software package, version 10.71^25,26^, developed at the Massachusetts Institute of Technology (MIT), the Harvard-Smithsonian Center for Astrophysics, Scripps Institution of Oceanography (SIO), and Australian National University.

We analysed the stations located on Stromboli volcano (SPLB, SPLN, STDF, SVIN) that are part of a cluster processing that includes 41 continuous GNSS stations located in North-eastern Sicily and the southern Calabrian Arc. We also included in the processing the GNSS station EIIV, located in Catania, on the roof of the Osservatorio Etneo (INGV), and some high-quality IGS stations (BOR1, CAGL, GLSV, GRAS, GRAZ, LROC, MATE, NOT1, NOTO, POTS, VILL, WSRT, WTZR, ZIMM), in order to improve the overall configuration of the network and to tie the local stations to an external global reference frame. We used double-differenced, ionospheric-free linear combinations of phase observations. The GAMIT solution was automatically iterated three times by applying a weighted least-squares algorithm, to reduce the residual of the estimated coordinate with respect to the a-priori values. GPS phase data were weighted according to an elevation-angle-dependent error model based on the actual scatter of the residuals from each station. Ambiguities were resolved using the Melbourne-Webbena wide-lane linear combination of phase and code. We also fixed the satellite orbits using the IGS final orbits (https://igs.org/products/) and applied second-order ionospheric corrections using the IONEX files from the CODE (Center for Orbit Determination in Europe).

The tropospheric delay was estimated using the Vienna Mapping Function (VMF1)^27^ and a 10° cutoff angle was fixed. The Earth Orientation Parameters (EOP) were constrained to a-priori values obtained from IERS Bulletin B and the ocean tidal loading was estimated using the Finite Element Solution (FES2004) model^28^. For diurnal and semidiurnal solid Earth tides, we used the International Earth Rotation Service (IERS) 2003 model. See Table 1 for a synthesis of the parameters adopted for GNSS data processing.

**Table 1:** Parameters adopted for GNSS data processing.

GNSS Processing parameters	
Elevation mask	10°
GPS orbits	IGS final orbits
Ocean tides loading	FES2004 model
Tropospheric mapping function	VMF1
Ionospheric correction	Second-order ionospheric correction
Solid earth tides	IERS2003 model
Earth orientation parameters	IERS Bulletin B
Reference frame/system	IGb14

**Fig. 2:**
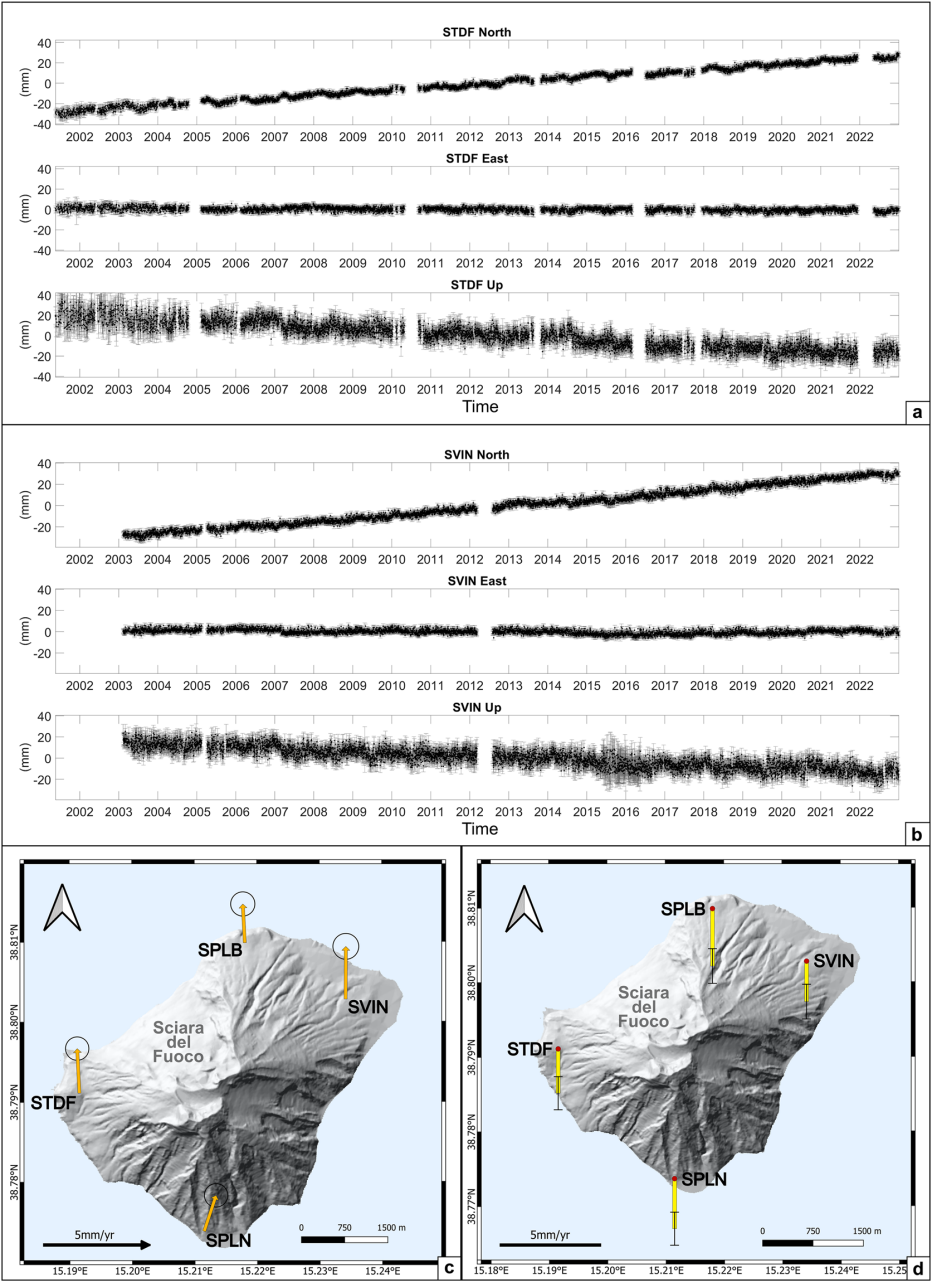
(**a,b**) North, East and Up components of the STDF and SVIN GNSS stations, respectively; (**c**) GNSS horizontal velocities (yellow arrows) with 95% confidence ellipses; (**d**) GNSS vertical velocities (yellow bars indicate subsidence) with 95% confidence error bars. Velocities are computed assuming a fixed Eurasian reference frame^29^, over the period from 2001 for SPLB, SPLN and STDF stations and from 2003 for SVIN, until 2022.

In the second step of the processing, the loosely-constrained solutions generated by GAMIT (h-files) were aligned to the IGb14 reference frame through the GLOBK/GLORG module^25^. Reference frame constraints were applied to each daily solution by means of a Helmert transformation. For each day, 6 free parameters (3 translations and 3 rotations) were estimated to minimize the deviations between the unconstrained solution coordinates and the fiducial station coordinates previously mentioned. We then obtained the 3 components (North, East and Up) of the time series position of each site in the IGb14 reference frame, rotated in the Eurasia-fixed frame^29^. An example is shown in Fig. 2.

Through GNSS time series analysis, we estimated and corrected the offsets due to instrumental changes or technical operations to the station sites (Table 2) using the Tsview interactive Matlab® based software^30^. Furthermore, we computed the velocity field with respect to IGB14 and Eurasian-fixed reference frame^29^. Velocities do not account for the transients due to volcanic activity. Horizontal and vertical velocities at each station with associated errors in IGb14 and Eurasian-fixed frames are shown in Table 3. Velocities in the Eurasian-fixed frames are shown in Fig. 2. Vertical velocities show a general subsidence with a mean value of 2.4 ± 0.2 mm/yr.

**Table 2:** Epoch and magnitudes of the offsets due to instrumental changes or technical operations to the station sites.

Site	Offset date (YYYY MM DD)	Offset values (m)		
		North	East	Up
SPLB	2005 05 07	0.00705	0.00926	0.00179
	2012 09 11	0.00234	−0.00642	0.01849
	2017 03 09	−0.00023	0.00024	0.00525
SPLN	2008 11 01	0.01106	−0.01622	0.01283
	2019 07 31	−0.00909	0.00018	−0.00349
STDF	2008 09 04	0.00324	0.00482	0.01274
	2010 10 22	−0.01009	0.00266	−0.00248
	2019 07 16	−0.00400	−0.00888	−0.00226
SVIN	2022 06 10	−0.01097	−0.00480	−0.00291

The values of the offsets have been estimated for each component (North, East, Up).

**Table 3:** Site code and North, East and Up velocity components (in mm/yr) and associated errors in IGb14 and Eurasian-fixed frames^29^.

Site	North	East	Up
Eurasia-fixed			
SPLB	1.92 ± 0.3	−0.1 ± 0.3	−2.85 ± 0.43
SPLN	1.78 ± 0.29	0.59 ± 0.29	−2.54 ± 0.41
STDF	2.21 ± 0.29	−0.11 ± 0.29	−2.2 ± 0.41
SVIN	2.61 ± 0.32	0.01 ± 0.32	−2.01 ± 0.43
IGb14			
SPLB	17.80 ± 0.33	22.75 ± 0.34	−3.73 ± 0.50
SPLN	17.57 ± 0.32	22.65 ± 0.33	−3.98 ± 0.48
STDF	17.36 ± 0.32	22.56 ± 0.33	−3.74 ± 0.48
SVIN	17.79 ± 0.32	22.57 ± 0.33	−3.61 ± 0.48

### Tiltmeters

#### Instrument

At Stromboli, continuous monitoring of Earth’s surface tilt variation began in 1992. Until the early 2000s, the tilt network consisted of biaxial analog tiltmeters installed inside boreholes at depths down to 3 meters (AGI Mod. 722 with a sensitivity of 0.1 µrad). Originally, the network was designed with three stations (Fig. 1), installed at Punta Labronzo (PLB), Timpone del Fuoco (TDF) beyond the southern and northern margins of Sciara Del Fuoco, and Punta Lena (PLN), with data transmitted in real time to Catania^11^. However, PLN and TDF were affected by site and instrumental problems, so PLN was subsequently decommissioned and TDF was reinstalled in 2011 at a new site, using a new high-resolution (10^−8^-10^−9^ radians) self-levelling digital tiltmeter with an on-board magnetic compass (Lily Model from AGI-Jewell with a sensitivity of 0.005 µrad) positioned in a 27 m deep borehole. The Lily has greater stability than previous instruments allowing long-term measurements and has its own memory for data storage; it is equipped with a motorized system capable of tilting the sensors up to ±10 degrees and works on a dynamic range of ±330 µrad. Upgrading the original sensors resulted in an improvement of about one order of magnitude in signal-to-noise ratio on the acquired data allowing to have signals with ca. 0.01 µrad of resolution. The PLB station, on the other hand, has not been active since May 25, 2022 due to a fire that destroyed the station, but will soon be reinstalled with a new generation sensor in a deep hole.

Borehole bubble tiltmeters are the most commonly used technique on volcanoes: the tilt sensor consists of a glass case partially filled with a conductive fluid inside which a bubble is suspended, that remains stationary with respect to the vertical gravity vector^31^. Its operation is based on the elementary physical principle that if a container is filled with liquid and an air bubble left in it, it will always be at the highest point of the container, since air is less heavy than liquid. In our case, the air-liquid boundary is in contact with two opposing electrodes, and its smallest displacements, in response to sensor rotation, are measured as changes in electrical resistance, which is a function of the angle at which the instrument is tilted. The electrical circuitry converts resistance values to angles through a conversion factor obtained by calibration of the instrument in the laboratory.

Each instrument is characterized by two axes that are generally oriented to detect a component, called radial (x-axis), positioned in the direction of the crater (positive values indicate uplift at the craters) and another component, called tangential (y-axis), in the orthogonal direction (positive values indicate uplift in the 90° counterclockwise direction relative to the radial). For the TDF station, tilt components are directed N275°E (tilt_x) and N185°E (tilt_y) respectively. The sensors (two tilt sensors and one temperature sensor) are located at the base of a cylindrical stainless steel body weighing about 7 kg, with a length of just under one meter and a diameter of about 5 cm, joined to the ground with fine quartz sand poured into the hole until it is almost completely filled.

#### Tilt data time series

**Fig. 3:**
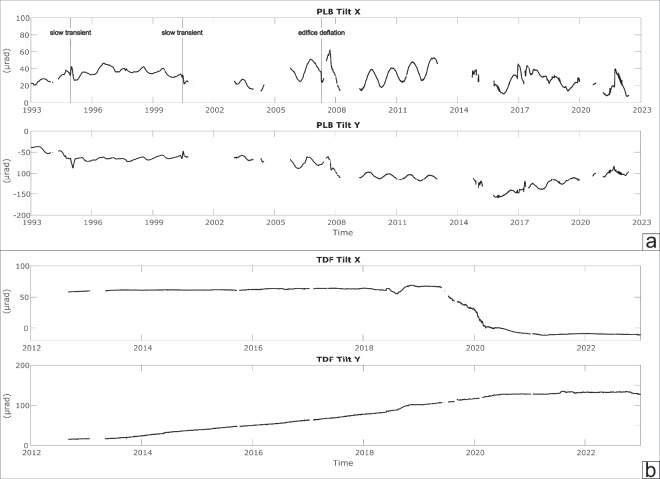
Time series recorded at the PLB tilt station from 1993 to 2022 (**a**) and at the TDF tilt station from 2012 to 2022 (**b**). We report tilt changes recorded along the two components for both stations (x-axis and y-axis).

Over the last 30 years, tilt networks have been expanded and improved: the development of highly performing instruments such as the Lily tiltmeter has made it possible to achieve much greater sensitivity than in the past, which allows the slow deformations of the ground to be studied much better by exploiting the variation in the inclination of the ground. Furthermore, thanks to the use of data storages which are now able to store an enormous quantity of data, in recent years it has been possible to move from the acquisition of data every 30 minutes (as was done until the early 2000s) to one sample per minute and in some cases even per second. The development of increasingly precise mathematical codes allows filtering and removing from thermoelastic and tidal effects, making the acquired data clearer. Taken together, all these aspects allow us today to investigate very fast volcanic phenomena (paroxysms and particularly intense explosive activity). In detail, the tilt analysis highlighted some types of peculiar events recorded on Stromboli (Fig. 3):

- slow transient anomalies (months) of a few tens of µrad, recorded at the PLB between 1994 and 1995^7^ and in May-July 2000^32^ which were modelled and interpreted as propagation of dyke intrusions.
- slow changes in inclination (weeks) during the 2007 eruption which recorded the continuous deflation of the volcanic edifice during the effusive phase which, with GPS data, allowed us to deduce a vertical source of depressurization, 3.5 km below the summit craters^33,34^.
- very small variations (about 0.1 µrad) detected about 2–4 minutes before the paroxysms, and rapid variations (1–2 µrad) during the phenomenon as on July 3, 2019^15^.

### Volumetric strainmeters

#### Instrument

Starting in 2006 on the island of Stromboli, two volumetric strainmeters, also called dilatometers, were installed on the north-eastern side of the island, near the village of Stromboli (SVO dilatometer), and in the south-western part, just north of Ginostra village (TDF dilatometer). Both sensors were cemented at the base of boreholes reaching a depth of 120 m. After the installation, comparison of data from both dilatometers for low‐ and high‐frequency signals (Earth tides and summit explosions, respectively) revealed that the TDF instrument was not well-coupled with the surrounding rocks, while the SVO dilatometer is recording precious data of Stromboli’s activity, yielding insights into eruption dynamics^10^ and small changes in the volcano behaviour prior to paroxysmal eruptions^16^.

Sacks-Evertson dilatometers are stainless steel cylinders, approximately 7 cm in diameter and 4 m in length, filled with degassed silicone oil, delivering two distinct signal outputs derived from two hydro-mechanical amplification systems, each consisting of a bellows-transducer-valve assembly. The first system comprises a larger sensing volume connected to a small bellow, the length of which varies in direct proportion to the volume of oil entering or exiting from the sensing volume due to its deformation. The position of the top of the bellows undergoes measurement through a linear variable differential transformer (LVDT). The second amplification system features a less sensitive assembly linked to the first one. The high-sensitivity output seamlessly integrates volumetric changes in the strained reservoir; meanwhile, the low-sensitivity output engages with the strained reservoir exclusively during rapid and intense strain changes, to prevent overpressures in the sensing device from causing permanent damage to the instrument. Usually, the low-sensitivity channel monitors pressure within a closed cell, a metric directly proportional to local temperature, with a temperature resolution of approximately 10 microdegrees. To keep the dilatometer within its operating range over indefinite time intervals, the two valves contained in the instrument are directly connected with the sensing volume and with the reservoir decoupled from the strain field. The first valve is opened for a few seconds as needed to allow oil to flow to or from the reservoir, while the second valve allows oil to flow between the second bellows and the unstressed reservoir. During normal measurement operations, both valves remain closed and open only when the cumulative strain approaches the instrument’s measurement limit. As electromechanical valves, they need to be electrically actuated, which implies that, to prevent damage to the instrument in the event of a power failure, these valves must be opened beforehand. When the supply voltage drops below the minimum operating value, the control electronics initiate a shutdown procedure to open both valves.

Each instrument is equipped with an external barometer for simultaneous air pressure measurement to remove spurious strain variations that might affect recorded data.

The nominal resolution of the Sacks-Evertson dilatometers stands at about 10^−11^, accompanied by a nominal dynamic range spanning from 10^−11^ to 10^−3^. Calibration for low-frequency signals in the installed strainmeters is achieved through meticulous comparison with Earth tides^35^, while higher frequency calibration is obtained through comparison with teleseisms or local seismicity^36^. Strain data are recorded and sampled at a rate of 50 samples per second using a proprietary datalogging system. Notably, Sacks-Evertson dilatometers’ dynamic range spans about 140 dB. Installation of Sacks-Evertson strainmeters in boreholes entails using expansive grout to couple them to the surrounding rocks: once the sensors are installed, they are impossible to be retrieved.

The borehole sensor is controlled by surface electronics, divided in two primary components: the analog electronics, which hosts the A/D converters, and the digital electronics also containing the control logic. These two components, along with the LVDT transducers contained onboard the sensor itself, implement a feedback control system that allows the instrument to always operate under optimal measurement and safety conditions.

#### Strain data time series

Strain data recorded by Sacks-Evertson strainmeters, due to the high dynamic of the instrument and since its output responds to input over a wide frequency range, are prone to be affected by anthropic noise, changes in atmospheric pressure, tides, rainfall, underground water movements, changes in underground temperature, earthquakes, as well as other crustal movements. Several kinds of procedures have been developed over time by geophysicists to remove the unwanted (“spurious”) signals from the actual recordings, in order to thereby obtain cleaner strain data, capable of representing the actual changes of the local strain in proximity to the installation site. The clearly most dominant signals in a strain data time series are associated with Earth tides and atmospheric pressure loading. Earth tides, due to the relative motion of Sun and Moon with respect to Earth, account for 10^−10^ strain over a frequency range of 10^−4^–10^−5^ Hz (periods of hours to days), and are induced by periodic, measurable forces: this allows a reproducibility of the phenomenon using numerical simulations software. On the other hand, atmospheric pressure, for its own characteristics, is a highly variable signal, spanning over extremely wide strain- and frequency-ranges. Both signals, however, are characterized by frequencies comparable with those of interest.

One of the most successful methods to remove tides and atmospheric pressure uses a combination of harmonic and non-harmonic techniques, through the implementation of Bayesian statistics (BAYTAP-G^37^). The software assumes that a given signal can be decomposed into a tidal component, a trend term, a perturbation due to an external source (an external force, like the atmospheric pressure, responsible for generating a change in the recorded signal) and some random noise superimposed. The main tidal components are hence calculated.

**Fig. 4:**
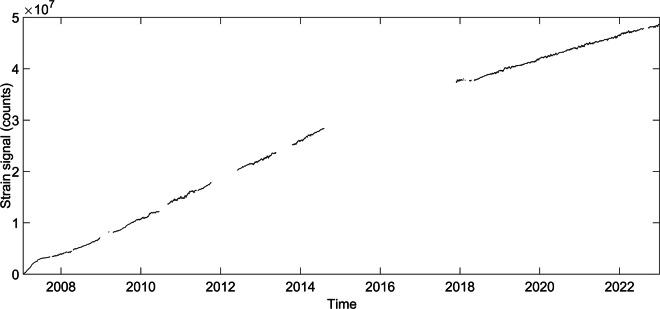
Time series recorded at the SVO dilatometer from 2007 to 2022: the overall trend shows the changes caused by the curing of the cement immediately after the installation (2007–2008 bump), as well as a general positive trend related with the plate tectonic local setting.

Data recorded through the integrated data-logging system, have been cleaned from all the outliers present (valve opening/closing, electronic spikes and other artefacts); then, using BAYTAP-G, tides and atmospheric correlation have been removed, using a semi-automated procedure developed in Matlab® (Fig. 4).

The analysis of strain data highlights the presence of events in conjunction of peculiar phenomenon recorded on Stromboli: dynamic strain transients^15,16^ as well as major and paroxysmal eruptions^9,10,13,38^.

## Data Records

### Tiltmeters and dilatometers data file format

The higher-rate data records presented in this manuscript, namely the datasets from dilatometers and tiltmeters, have been stored using ASCII files containing one time-series related to a specific sensor or gauge. Accompanying datasets (temperature and barometric pressure) are shared as well, since they are fundamental to improve data resolution, and the provided format is the same as the main time-series data.

The provided ASCII files are stored as two-columns datasets, containing information about the timestamp of each sample recorded, along with the corresponding data value.

### Data types

Due to their sampling rate, tiltmeter and dilatometer data files comprise a different number of samples, resulting in different file sizes: tiltmeter data were acquired using two different sampling rates in different time frames, being recorded with rates of 1 sample every 60 or 1800 seconds (1 or 30 minutes, respectively). On the other hand, the whole dilatometer dataset accompanying the present manuscript has been recorded at 1 sample per second.

The GNSS raw data that we processed are in the RINEX format, with a 30 s sampling rate and 24 hours (00:00:00 - 23:59:30 UTC) of continuous data from 2003/2004, while the data collected before 2003/2004 have shorter daily acquisition intervals (three or four hours). Here, we provide 3D coordinate time series on a daily basis.

#### GNSS dataset

The raw data recorded by the GNSS ground deformation monitoring network are available in the Receiver INdependent EXchange format^39^. The dataset contains also the station coordinates and the receiver, antenna and occupation-time information for each site. We provide processed data that include both unfiltered and filtered position time series of each GNSS station. Unfiltered time series are very useful for analyzing volcanic-induced ground deformation and for investigating volcanic processes as they contain transient signals, which are deviations from long-term linear motion due to volcanic activity. Also scattered data, which might appear as higher noise levels can provide insights into specific volcanic phenomena, as suggested by^40^. In the filtered position time series we removed outliers and corrected the instrumental offsets. They provide a comprehensive view of crustal motion, incorporating both secular signals and transient events.

The GNSS time series are provided in text ASCII format, the “pos” format^41^, realised by the Plate Boundary Observatory for GNSS time series solutions sharing, in the online repository^42^. They are stored in the “GNSS_Time_Series” folder. Within this folder, there are the two subfolders “GNSS_filtered” and “GNSS_unfiltered”, containing the filtered and unfiltered time series files, respectively, which have the following naming conventions: **XXXX_filtered.pos or XXXX_unfiltered.pos**where XXXX is the name of the permanent station (SPLB, SPLN, STDF or SVIN).

#### Tiltmeter dataset

The tilt dataset is composed by ASCII text files. The data file is made up by multiple columns, each containing information about the recorded data (please refer to section “Data Records” for more detailed specifications): first column contains the recording time of the sample, the second column reports the air temperature, third and fourth columns contain radial and tangential tilt components respectively, while the latter column reports the actual tiltmeter temperature. Since temperature will affect the inclinometer sensor, which could be the cause of fluctuations on the measurements, both temperature values can be used to reduce the uncertainty and obtain more reliable results.

#### Strainmeter dataset

Data are provided as two columns ASCII files. The recorded output from the high-sensitivity strainmeter hydro-mechanical amplification system, as well as from the local barometric pressure transducer, have been stored as equispaced ASCII data. The datasets were stored in the “Strainmeter_dataset” folder in the online repository^42^. Within this folder, there is a subfolder whose name refers to the strainmeter sensor. File names have the following convention:

**YYYYstrain.txt**

**YYYYbar.txt**where YYYY is the reference year. The former file name refers to data recorded from the high-sensitivity hydro-mechanical amplification system, while the latter contains data collected from the barometer installed near the dilatometric sensor. Both strain and barometric data are stored at 1 sample per second.

## Technical Validation

### GNSS

The daily GNSS position time series were analysed in order to estimate annual and semi-annual components, noise and remove outliers. We manually selected offsets due to instrumental changes and estimated the values for each offset using the Matlab® based interactive software Tsview^30^.

To estimate annual components, semi-annual components, noise, and remove outliers, we used Hector software based on maximum likelihood estimation (MLE)^43,44^. The Hector software enabled identifying outliers in the time series automatically, based on the interquartile range (IQR) of the residuals of the linear least squares fit. We removed from the time series the residuals outside the chosen threshold (IQ factor multiplied by IQR) that were considered outliers. The value we used for the IQ factor is 2.

To obtain a realistic estimate of uncertainty, it is necessary to know the properties of noise on GNSS position time series that can be also influenced by transient events with a preferential geographical orientation^45^. White noise uncorrelated with time underestimates uncertainties on velocities, as shown by several works^46–52^. The best way to describe noise in GNSS position time series is to combine white noise with time-correlated noise^53^. We accounted for uncertainty in our data and analysed the position errors of the sites using Hector software^44^, which allowed us to obtain insights into the properties of noise on the time series by combining the generalized Gauss-Markov noise model with white noise in order to obtain a realistic estimate of daily positions^53^.

The Normalized Root Mean Squares (NRMS) and Weighted Root Mean Squares (WRMS) values of all time-series were estimated using GLOBK software after correcting offsets, estimating annual components, semi-annual components, noise and after removing outliers (Table 4).

**Table 4:** Normalized Root Mean Squares (NRMS) and Weighted Root Mean Squares (WRMS) values for the three components of every time series estimated by GLOBK software.

SITE	NRMS	WRMS (mm)
SPLB_North	1.07	2.2
SPLB_East	0.92	1.6
SPLB_Up	0.77	4.4
SPLN_North	0.93	2.2
SPLN_East	0.88	2.3
SPLN_Up	0.76	6.0
STDF_North	1.03	1.9
STDF_East	0.92	1.8
STDF_Up	0.80	5.0
SVIN_North	1.02	2.1
SVIN_East	1.01	2.0
SVIN_Up	0.76	4.5

### Tiltmeters

**Fig. 5:**
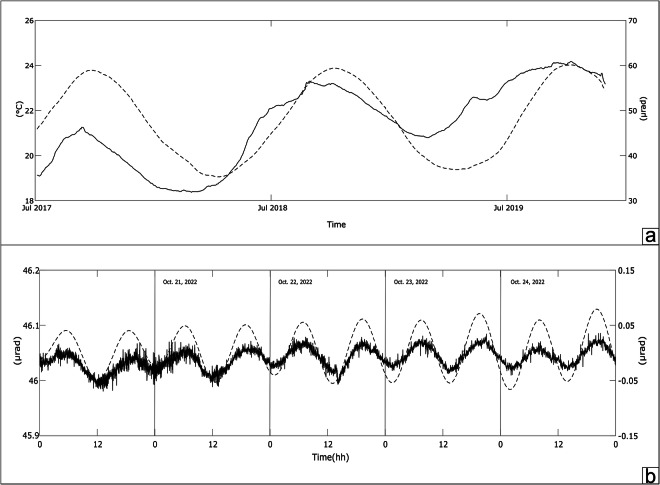
PLB raw tilt signal (tangential component in solid line) and ground temperature (3 metres depth in dashed line) recorded in 2017–2019. Tilt fluctuations are caused both by thermic and thermoelastic effects (**a,b**) TDF N°275°E component (solid line) compared with theoretical Earth tide (dashed line) obtained by using ETERNA 3.4 software^61^. A tidal amplitude factor of 0.7 was applied to the theoretical signal^62^.

Borehole bubble tiltmeters use high precision electrolytic sensors, calibrated by the manufacturer, to measure angular movements. These instruments are affected by noise caused by several meteorological factors but mainly by temperature fluctuations (e.g.^54,55^). Temperature changes affect sensors (thermic effect) for about 1–2 µrad/°C^56^ and cause thermoelastic effects, i.e. real ground movements^57,58^. Stromboli PLB station used an AGI 722 model tiltmeter with a precision of ca 0.1 µrad, installed at 3 metres depth. In these conditions, temperature shows seasonal changes of 4–5 degrees, producing annual tilt variations of about 15–20 µrad (Fig. 5a).

However, if tilt sensors are positioned in deeper holes (20–30 meters), where temperature is stable, this allows accurate recordings with a very high signal-to-noise ratio^59^. In the last 15 years, several deep stations have been installed on Sicilian volcanoes by using advanced high resolution (5 nanoradians), self-levelling instruments (LILY by AGI/Jewell and Pinnacle 5000 by Pinnacle/Halliburton) that has enabled acquiring signals with a very low noise showing tide components^31,60^. TDF is positioned at a depth of 27 metres, shows a stable ground temperature of 21 °C and records high resolution signals (ca. 0.01 µrad) modulated by diurnal and semidiurnal Earth tide components with an amplitude of about 0.1 µrad (Fig. 5b).

The recorded TDF signal compared with computed earth tides tilt carried out with Eterna 3.4^61^ software package shows a good correlation albeit with a smaller amplitude. However, tidal models based on an elastic Earth predict higher amplitudes with respect to the observed signals^62^ also depending on local conditions^31^.

### Strain data

The technical quality of the dataset has been validated by comparing strain data with synthetic signals and recorded seismometers and barometers data.

High instrumental sensitivity of dilatometers for earth deformation monitoring has an effect on the data accuracy which depends on installation and/or environmental factors like the quality of the surrounding rocks, the coupling instrument-medium, local ground-water flow, pore pressures induced by rainfall, temperature changes, change in barometric pressure and anthropic activities. For these reasons the response of the instrument cannot be established in the laboratory but must be checked with appropriate *in situ* calibration, once the installation is completed. Such calibration is made by comparing the recorded strain with estimated reference signals such as the ones produced by diurnal tides, or by the travelling waves of teleseismic events.

The SVO strainmeter is installed in competent rock that is below sea level: due to sea water infiltration in the borehole, water temperature changes dominate the apparent tidal strain, which in turn precludes tidal calibration at the site^8^.

**Fig. 6:**
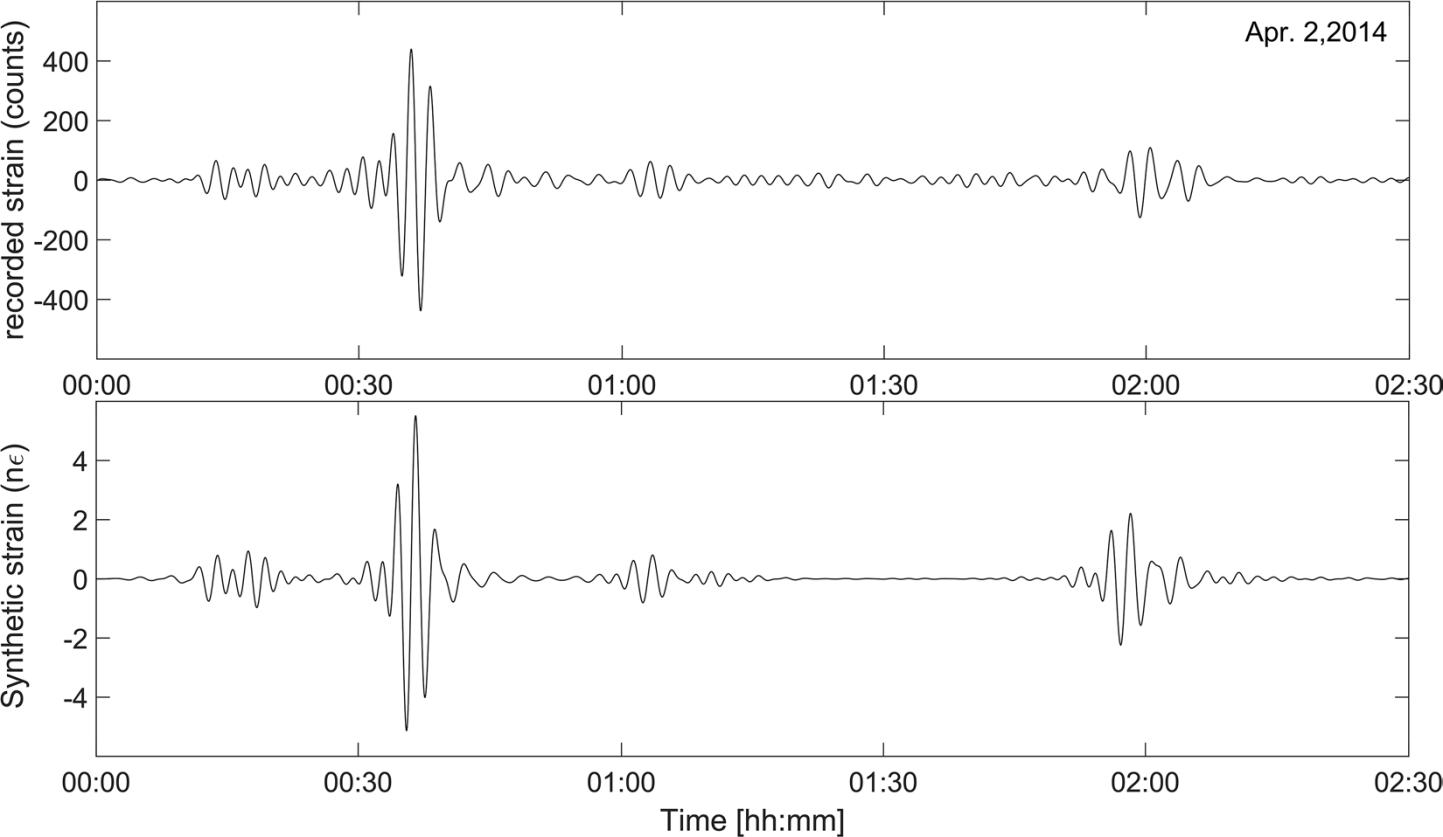
Calibration through the comparison of recorded and synthetic signals for the Iquique, coast of Chile, MW 8.1 earthquake on April 1, 2014. Synthetic strain is provided by the Kamigaichi code. Both signals are filtered in the 100–200 s period.

Our calibration approach relies on comparing the dynamic strain amplitude of long-period surface waves from very energetic teleseismic events with calculated synthetic strain waveforms. The Fortran code for the calculation of normal-mode synthetic seismograms is provided by O. Kamigaichi (Japan Meteorological Agency). The synthetic strain was computed from the Normal Modes Theory for the Earth oscillations^63^ using the preliminary reference Earth model (PREM) for the Earth medium properties^64^, and as input the centroid moment tensor (CMT) solution^64^. The comparison between recorded and normal-mode synthetic signals both filtered in the period band 100–200 s, for several teleseismic events, allowed us to calibrate the dilatometer data. Figure 6 shows the result for the Iquique, coast of Chile, MW 8.1 earthquake on April 1, 2014: in the analysed frequency range, the similarity between the synthetic and recorded signals is very good.

The result of our analysis, obtained by the best overlap of the signals, provided 10^−2^ nε as the calibration factor.

The Stromboli dilatometer has shown the capacity to detect short- (several minutes) and medium-term (days to weeks) precursors^10,15,16,38^ capable of forecasting an incoming paroxysmal eruption, as well as strain variation in concomitance with major explosions^9,13^.

The large spectrum of detected signals^24^ reveals the high-sensitivity of strain measurements spanning time-periods from seconds (seismic processes) to months (long-term tectonic and environmental processes). Recording of strain waves provides a large range of applications in volcanology: they have been used in estimating seismic phase velocities^65^, modelling earthquake source processes^66,67^, detecting Earth free oscillations^68^, and focusing our attention on the dynamic strain recordings, in the detection and characterisation of atmospheric infrasound generated by earthquakes^69^.

**Fig. 7:**
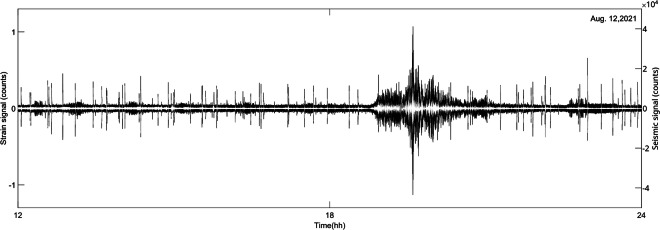
Comparison between the vertical component of seismic record (white line) and strain signal (black line) of August 12, 2021, in correspondence of a teleseismic event: there is a good similarity in the seismic frequency range.

In order to evaluate the Stromboli strainmeter detection capacity of these signals, the strainmeter data are compared with the recordings of seismometers and barometers in coincidence with particular events. Figure 7 shows the strain and velocity recordings in correspondence of a teleseismic event and ordinary strombolian explosions: within the seismic range, the two signals correspond.

**Fig. 8:**
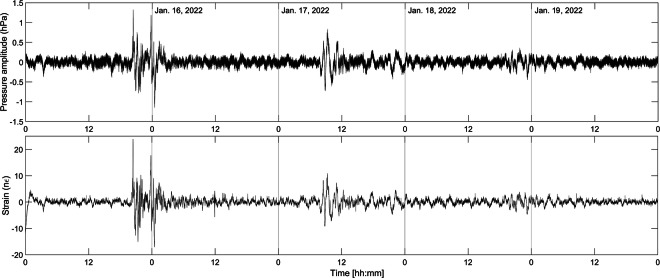
Comparison between the atmospheric pressure (upper) and strain signals (lower) produced by the eruption of the Tonga–Hunga Ha’apai volcano which occurred on January 15, 2022. The overlapping of the pressure waves with the recorded strain is flawless.

The major eruption of the Tonga–Hunga Ha’apai volcano which occurred on January 15, 2022 produced several atmospheric disturbances at a global scale, in particular a shock-wave pressure wave travelling in the troposphere and well visible in the Mediterranean area^70^. The recorded fluctuations of atmospheric pressure travelled around the Earth as a Lamb wave, with a frequency ranging between 0.3 and 10 MHz composed of acoustic and gravity waves. The Fig. 8 shows the perfect overlapping of the pressure waves with the recorded strain.

## Data Availability

No custom code was generated for this paper.
